# Analysis of the Causes of Damage to the Steel Drive Shaft Used in a Paint Mixer

**DOI:** 10.3390/ma18204798

**Published:** 2025-10-21

**Authors:** Wojciech Skotnicki, Dariusz Jędrzejczyk

**Affiliations:** Faculty of Mechanical Engineering and Computer Science, University of Bielsko-Biala, Willowa 2, 43-309 Bielsko-Biala, Poland; djedrzejczyk@ubb.edu.pl

**Keywords:** FEM analysis, drive shaft, surface treatment, damage analysis

## Abstract

This article presents an analysis of the causes of damage to the shaft used in a paint mixer (made of 1.0501 steel, with diameter ø = 90 mm and length l = 3451 mm). The observed damage occurred in both the shaft before regeneration and the part regenerated by surfacing. The initial analysis consisted of both macroscopic and microscopic observations of the shaft cross sections. Additionally, hardness measurements were made using the Vickers method (HV0.1). The results of microstructure observations were used as the basis for further finite element analysis (FEA). The FEA simulations made it possible to identify the places most susceptible to damage and assess the stress distribution during the shaft’s application. Based the FEA results, in order to improve the durability of the analyzed structural element, changes in the shaft geometry and the use of different chemical steel compositions are proposed.

## 1. Introduction

Although very sophisticated construction materials are used in the production of drive shafts, metal shafts are still the most commonly used in the machinery industry. A serious problem in the operation of machines is various types of failures, the causes of which can be caused by many factors. One of the most important factors is improper use or excessive load on the machine, which leads to excessive wear and damage to the steel components. In addition, inadequate maintenance, lack of regular inspection and technical maintenance can contribute to failures [1].

Other potential causes of failure are material defects, such as inhomogeneities in the steel structure, the presence of impurities, or material discontinuities on a macro- and microscopic scale. Such defects can lead to a reduction in the strength of the steel and an increased risk of cracks. In addition, environmental factors, such as corrosion, high temperatures, and chemical or weather conditions, can adversely affect the mechanical strength of steel, contributing to reduced durability and consequently to machine failure [2,3].

Fatigue fracture is the most common failure mechanism in shafts and is often directly attributed to geometric design errors [4,5]. To prevent such failures, stress concentrations resulting from local changes in shape should be carefully avoided during the design process [6,7,8,9,10,11,12]. In addition to high stress concentration, the primary factors contributing to crack initiation and propagation include structural discontinuities caused by small radii in transition sections, as well as surface roughness [13,14,15,16].

The production process of metal drive shafts involves multiple stages—from machining to heat treatment—each of which can directly or indirectly influence the mechanical and operational properties of the shafts, as well as their susceptibility to failure [17,18,19,20]. One significant risk arises during chemical treatment, particularly during surface cleaning (pickling), where the phenomenon known as hydrogen embrittlement can occur [21]. Similarly, improperly conducted heat treatment—such as hardening—may result in the formation of excessively brittle martensite, promoting microcrack formation and significantly reducing the service life of the shaft [22]. The selection of material for the drive shaft is a very important stage in the design process. On one hand, the material chosen for the shaft should be as inexpensive as possible, but generally, cheaper materials tend to have lower mechanical properties and poorer quality. On the other hand, choosing a more expensive material with better mechanical and quality characteristics allows for the production of a shaft with smaller dimensions (lighter), which also makes it possible to select smaller (and cheaper) bearings. The choice of material is especially important in the case of heat-treated shaped shafts, as these shafts usually operate under variable loads. Factors influencing the material selection include required strength, stiffness, the purpose of the axes and shafts, and the product’s cost. Availability of the material and the intended application of the drive shaft—such as in the aerospace industry, automotive transport, air transport, sea transport, or heavy industry—are also crucial considerations [23,24,25,26,27].

A wide variety of materials are used to make drive shafts in different applications. The choice of material for drive shafts significantly impacts their performance, durability, weight, and cost. Steel remains the most widely used material for drive shafts due to its excellent strength, toughness, and availability. High-strength alloy steels, such as chromoly (chromium–molybdenum) steels, are popular because they offer a good balance of strength and ductility, allowing drive shafts to withstand high torque and torsional stresses [4]. Steel shafts are also relatively cost-effective and easy to manufacture. In addition to carbon steel, various types of steel are used for the production of drive shafts of industrial mixers, including paint mixers: in equipment for the paint, chemical, and nitrogen industries (1.4301, 1.4541); in the textile, chemical, and food industries (1.4571); for devices with wall thickness above 20 mm to be operated in environments exposed to intercrystalline corrosion and some aggressive chlorines (1.4404); for the textile and paper industries (1.4436); for parts to be operated in environment containing sulfuric, phosphoric, and formic acids (1.4539); and for parts for machines for the chemical and petrochemical industry to be operated under high mechanical loads in strongly corrosive environments and chlorines (1.4462–Duplex) [28]. Aluminum alloys or aluminum–composite materials are favored in applications where weight reduction is crucial, such as in sports cars and racing vehicles [29,30].

Recent advancements have seen the use of composite materials, such as carbon fiber-reinforced polymers (CFRP), in drive shafts. These materials provide exceptional strength-to-weight ratios and excellent fatigue resistance [31,32,33].

In modern manufacturing, machine components—including drive shafts—are designed and validated using specialized software, such as finite element method (FEM) tools, which enable advanced modeling and analysis [34,35,36,37].

Obviously, not every shaft needs to be taken out of service after it has been damaged. Shaft repair technologies can include a variety of methods and techniques (welding, surfacing, metalizing, metal-epoxy surfacing, plating, sleeve), depending on the type of damage, shaft material, and application requirements [38].

Welding can be used to repair non-heat-treated steel shafts in easily accessible areas [39]. Repair welding of shafts is performed to remove various defects of drive shafts: abrasive wear damage; adhesive wear; corrosion damage, mechanical damage to the surface—recesses, grooves and cracks. In special cases, gas tungsten arc welding (GTAW) is used (steel shafts of turbochargers of automotive engines) [40,41,42].

This research was carried out in order to explain the causes of damage to a paint mixer shaft during operation. The damage was located in the part of the threaded connection and in the part regenerated by surfacing. In order to analyze the reasons for the damage, macroscopic, microscopic, and metallographic tests were carried out along with measurement of the hardness on the cross section of the tested shaft. On the basis of the results obtained and the FEM analysis, design solutions are proposed to increase shaft lifetime without additional significant financial outlays.

## 2. Materials and Methods

**Figure 1:**
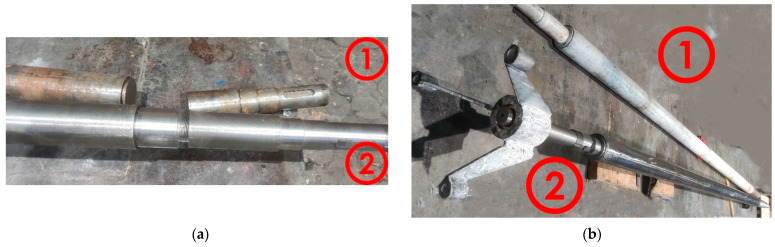
Appearance of the damaged and the new shafts (1—damaged shaft, 2—new shaft prepared for installation): (**a**) the place of fracture; (**b**) appearance of the entire shaft.

This paper presents results of tests of a drive shaft used in a paint mixer and damaged during operation (Figure 1). The shaft was made of 1.0501 steel. The chemical composition of the steel based on the PN-EN 10083-2 standard [43] and chemical analysis is presented in Table 1.

**Figure 2:**
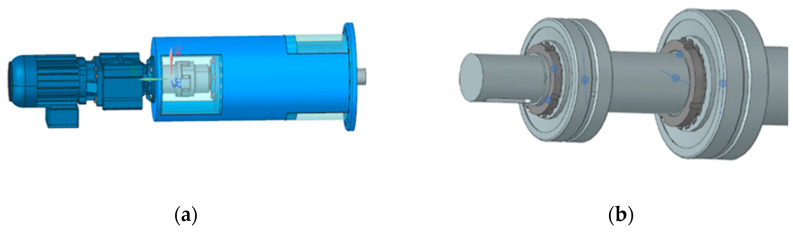
Visualization of the drive unit (**a**) and shaft bearing (**b**).

**Figure 3:**
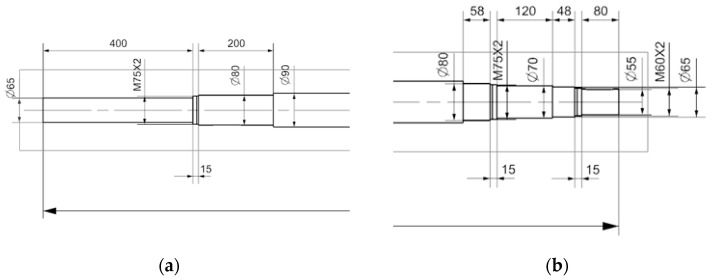
Dimensional details of the shaft necks at the bearing point (**a**) and at the bearing arrangement in the bracket (**b**).

The shaft (ø = 90 mm; length = 3451 mm) is an integral part of the paint mixer drive unit. The drive unit consists of a Nord SK572 gearmotor generating a torque of 3800 Nm connected by a ROTEX 48 coupling. The upper bearing of FAG 22313 E1 C3 is secured with a lock nut KM12 and the lower bearing of FAG 22316 E1 XL with a lock nut KM15. The shaft is mounted in a bearing console in two double-row self-aligning bearings and stabilized at the bottom of the tank with a bronze shell. Visualization of the drive unit and shaft bearing is presented in Figure 2. Dimensional details are presented in Figure 3. During the surfacing process performed by welding, the following parameters were applied to ensure optimal metallurgical properties and process stability. A welding wire with a diameter of 1.2 mm and direct current electrode-positive (DCEP) polarity was used, meaning the positive pole was connected to the wire electrode. The arc voltage was set to 25 volts, while the welding current reached a value of 300 amperes, indicating a relatively high-energy process suitable for effective material deposition. As for the shielding environment, a protective gas mixture consisting of 82% argon and 18% carbon dioxide was utilized to provide adequate arc stability and weld pool protection. The shielding gas was supplied at a flow rate of 22 L per minute, ensuring sufficient coverage of the weld zone to prevent contamination from atmospheric gases such as nitrogen and oxygen. These parameters collectively contributed to a stable arc, good weld bead formation, and proper metallurgical bonding during the surfacing operation.

The study of the causes of shaft damage included the following steps:

- Macroscopic studies of shaft fracture using a Delta Optical SZ-630B optical microscope (Delta Optical, Minsk Mazowiecki, Poland) with a magnification of 10×.
- Preparation of samples for microscopic observations: mounting the test specimens—Ecopress 100 device (Metkon INC., Mauldin, SC, USA); surface grinding—abrasive papers: 180, 500, 2000; surface polishing with diamond suspension—Scandimatic 33305 device (SCAN-DIA GmbH, Hagen, Germany); etching in 4% HNO_3_.
- Hardness measurements at the cross section of the subsurface layer of the drive shaft were conducted using a Vickers hardness tester (HV 0.1) and a Mitutoyo Micro-Vickers HM-210 A device (Model 810–401 D, Mitutoyo, Kawasaki, Japan) and Rockwell FENIX 300U device (Innovatest, Wiry, Poland). Microscopic examination of metallographic specimens was carried out using an Axiovert 100A optical microscope (Carl Zeiss GmbH, Oberkochen, Germany) with the use of the computer image analysis software ImageJ (version 1.54p) and an Axiocam 305 camera (Carl Zeiss GmbH, Oberkochen, Germany).
- FEM simulation—modeling and evaluation of stress distribution in individual shaft cross-sections, which identifies potential places most vulnerable to damage.

## 3. Results

### 3.1. Macroscopic Observations

**Figure 4:**
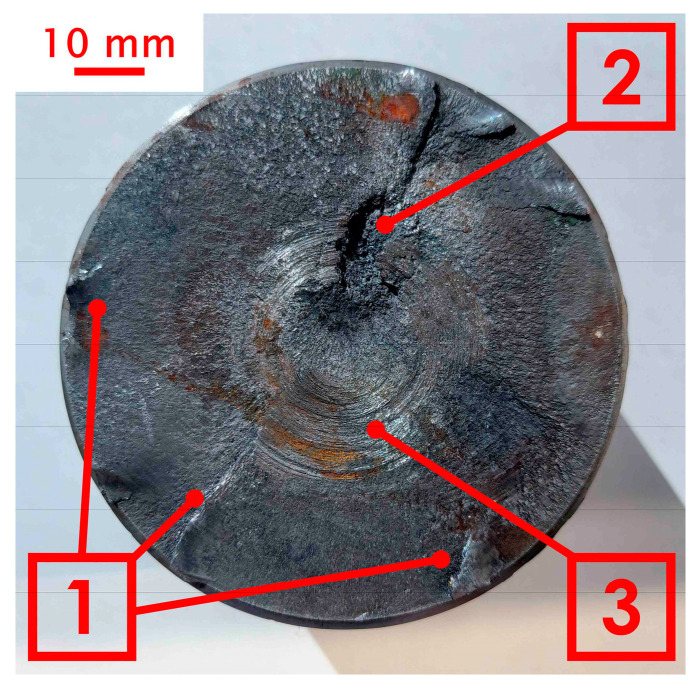
Appearance of the analyzed drive shaft fracture (1) ratchet marks, (2) fracture zone, (3) finishing.

Macroscopic observations were carried out on the surface of the obtained fracture. During 20 years of operation, the wear of the shaft was manifested in its abrasion and loss of material on the surface. After this period, the shaft was regenerated by surfacing. For surface welding, BD-2.300 wire was used (C 0.14; Si 0.50; Mn 2.0; Cr 2.5; M0 0.3) with a hardness after welding in the range of 280–325 HB [44], and then shaft was ground. The following parameters were used during surfacing by welding: wire diameter 1.2 mm, polarity on the wire positive, arc voltage 25 V, current 300 A, shielding gas argon 82% + CO_2_ 18%, gas flow 22 L/min. Three years since regeneration, critical damage to the shaft occurred—a through crack. The fracture visible in Figure 4 was created during the operation of the shaft as a result of the propagation of microcracks initiated near the outer surface.

**Figure 5:**
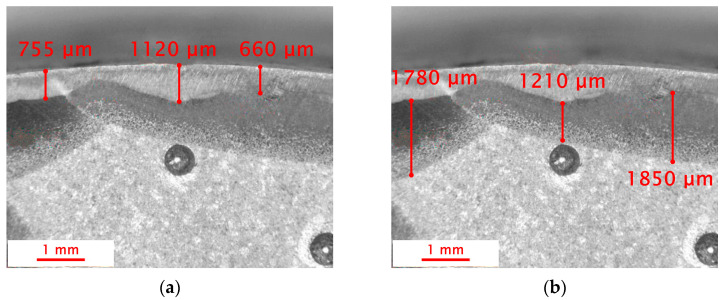
The subsurface microstructure observed in the cross section of the tested drive shaft: (**a**) results of overlay layer thickness measurements; (**b**) heat-affected zone (HAZ) thickness measurement results.

The crack initiation sites were located on the outer surface of the drive shaft, where crack seeds are visible. In the case of materials subjected to variable loading, cracks are initiated on the external surfaces, where various defects, scratches, micro-chipping, or material inhomogeneities may occur [45]. These defects can promote stress concentration, which contributes to the development/propagation of microcracks, leading to structural failure. In the macroscopic image, a small area of the final fracture zone (2) is visible. The presence of a clearly limited final fracture zone area indicates that the shaft may have fractured under a relatively small force, but with a high stress concentration in that specific area. Small final fracture zones suggest a sudden, dynamic failure, which may be caused by a high concentration of stresses or internal material defects that further weaken the material’s structure. The area labeled 3 indicates the fatigue fracture zone, where fracture smoothing occurs as a result of the rubbing and peening of the crack surfaces. This smoothing is evidence that during operation, relative movements took place between the parts of the shaft separated by the crack, causing them to rub against each other. This may be caused, for example, by increasing vibrations and oscillations. Figure 5 shows the structure observed using the Delta Optical SZ-630B stereomicroscope. The main purpose of the conducted observations was to analyze the geometry and assess the quality of the overlay weld layer. During the examination, the thickness of the heat-affected zone (HAZ) was also measured. The observations were carried out at a magnification of 10×.

The surfacing welding process results in the formation of several characteristic zones [46]. Macroscopic observations revealed that the surfaced layer was uneven, with a thickness ranging from 350 to 1100 μm. Such variations may result from several factors, including an insufficient amount of filler material supplied during the surfacing process, which can lead to an excessively thin layer in certain areas. Additionally, the improper selection of welding wire—in terms of both material type and diameter—can also affect the resulting layer thickness. Another factor that may influence the unevenness is the variable speed of the surfacing process. Surfacing that is carried out too quickly or too slowly can lead to uneven distribution of the material, resulting in inconsistent thickness of the deposited layer. The heat-affected zone (HAZ) also showed significant variations in thickness, ranging from 800 to 2000 μm. The thickness of this zone directly depends on the surfacing parameters, such as heating intensity, surfacing speed, and the surfacing technique used. An excessive amount of energy delivered to the material in a short period of time increases the thickness of the heat-affected zone (HAZ), which may lead to a greater tendency of the material to crack. Changes in surfacing parameters, such as surfacing speed, can also result in uneven heating and cooling of the material. If surfacing is performed irregularly, excessively rapid heating may cause local overheating, while uneven cooling can lead to the formation of internal stresses. Variations in the power of the heat source can also cause local fluctuations in surfacing intensity, which in turn lead to inconsistencies in the thickness of the deposited layer and in the heat-affected zone itself.

### 3.2. Microscopic Observations and Hardness Measurements

The next stage of the research was detailed microscopic observations of samples taken from the drive shaft (from areas located near the fracture zone) to accurately assess the material’s microstructure after the surfacing process. These studies provided information on the layout and distribution of the individual zones occurring near the surfaced layer. The microstructural evaluation was carried out using the Axiovert 100A optical microscope. Observations were conducted in etched cross sections in bright field. The images were recorded using the Axiocam 305 microscope color camera. Additionally the outer HAZ layer was verified by observation with an SEM application (Phenomworld, Thermo Fisher Scientific, Eindhoven, Holland).

**Figure 6:**
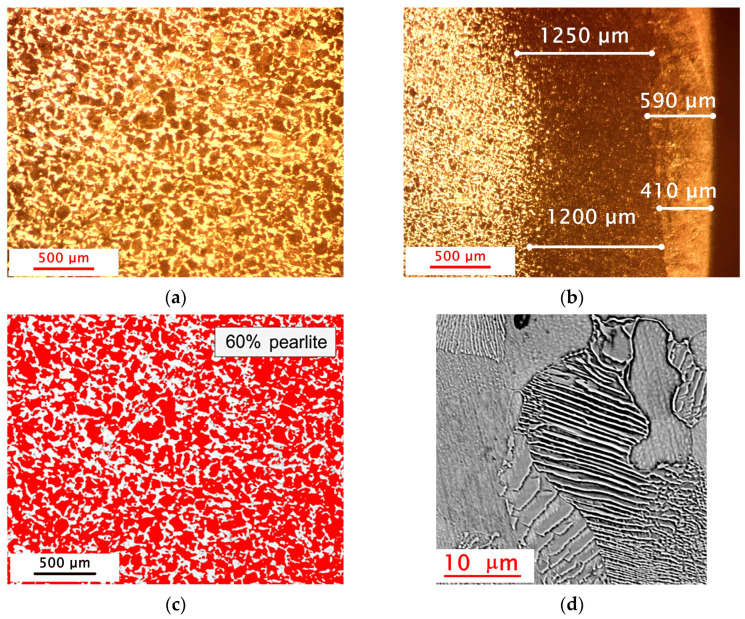
The microstructure observed in the cross section of drive shaft: (**a**) drive shaft core; (**b**) drive shaft outer subsurface, etched with HNO_3_; (**c**) ImageJ software analysis result—60% of perlite marked in red; (**d**) the fine pearlite microstructure observed in the outer HAZ layer.

The measurements of the thickness of the deposited layer and the heat-affected zone were carried out using ImageJ V1.54p. This software enabled precise determination of the dimensions of the deposited layers and the areas affected by heat, which is crucial for evaluating the effectiveness of the surfacing process and predicting the mechanical properties of the material. The results of the observations and measurements are presented in Figure 6.

During the microscopic observations, the microstructure of the steel was examined both in the core and in the subsurface layer to verify its conformity with the declared steel grade—1.0501. For this purpose, ImageJ software was also used to determine the proportion of pearlite and ferrite. Figure 6c presents the result of the pearlite content measurement (highlighted in red). A pearlite content of 60% corresponds to a carbon content of approximately 0.3% [47].

**Figure 7:**
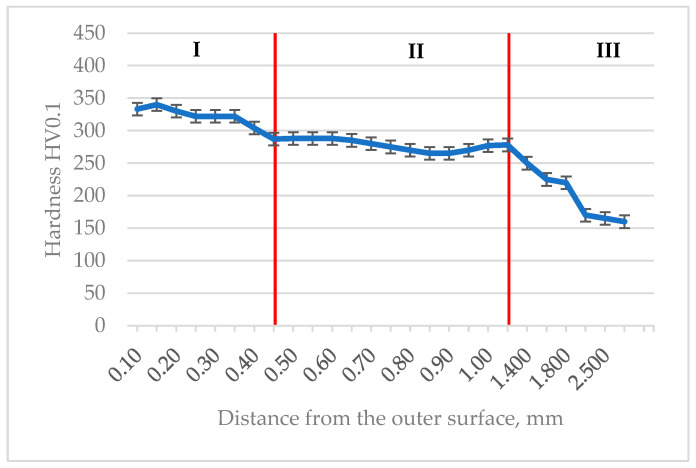
Change in hardness measured on the cross section of the tested drive shaft (I) overlay layer, (II) heat-affected zone, (III) core of the sample.

As a supplement to the metallographic studies, hardness measurements were carried out using the Vickers HV0.1 method, which allowed for the identification of individual microstructural phases (the hardness of carbon steel after heat treatment significantly depends on the carbon content [48] and the proportion of specific phases: martensite, bainite, troostite [49]). It is obvious that for a more precise analysis of the microstructure—particularly the phase distribution—a more advanced technique such as EBSD or XRD can be used [50]. However, in the present case, the hardness measurements, correlated with optical and SEM microscopic observations, appeared to be sufficiently accurate. The tests were conducted starting from the outer surface of the sample and continuing toward its core. Based on the measurements, the presence of three distinct zones was confirmed, with their extent corresponding to the results of previous macroscopic and microscopic observations as well as to literature data. The first zone, where the highest hardness was recorded, was located directly at the surface of the sample and corresponded to the deposited layer. In this zone, the average measured hardness value was 325 HV0.1, which is consistent with the manufacturer’s specification [44]. These values result from the introduction of materials with higher hardness (which can reach in some cases even up to 3000 HV) into the subsurface zone, which should provide improved surface resistance to wear and mechanical damage [51]. From the perspective of the specificity of repaired defects and the main causes of wear and tear in industrial machinery and equipment (about 50% of all defects result from wear, about 15% from adhesion, erosion and fretting approximately 8% each, corrosion about 5%, and the remaining 14% can be attributed to other processes [52,53]), increasing the wear resistance of the outer layer is of great importance. The second zone, located at a distance of 0.45 mm to 1.2 mm from the sample edge, was the heat-affected zone (HAZ). In this zone, the measured hardness averaged 275 HV0.1, which suggests that the process occurring as a result of surfacing led to a partial transformation of the material’s structure, forming a pearlitic structure with high dispersion (typical lamellar structure) (Figure 6d). Although the hardness in this zone was lower than in the deposited layer, it remained higher compared to the core of the sample. The third zone was the core of the sample, where the measured hardness values were the lowest, with an average of 220 HV0.1. This zone showed no influence from the surfacing process and retained its original hardness values, corresponding to a pearlitic–ferritic structure. The hardness measurement results are presented in Figure 7.

### 3.3. FEM Analysis of the Paint Mixer Shaft

The aim of the presented research was to conduct a detailed FEM analysis of the shaft load.

In particular, the analysis included:

- Identification of areas of potential torsion that may affect the structural integrity of the shaft and its ability to transmit torques.
- Assessment of the impact of various loads, both static and dynamic, on the strength and durability of the structure.

The analysis carried out formed the basis for assessing the structural safety and optimizing the design in terms of reliability and performance. The analysis was conducted using NX Siemens software version 2406 (formerly Unigraphics), which is one of the leading integrated CAD–CAE–CAM environments used in modern engineering industries [54,55].

The first stage of the FEM analysis was to conduct simulations of the loads on the drive shaft made of three grades of non-alloy steels with varying carbon content. The steel grades included in the study were: low-carbon steel, designation 1.0501 (PN-EN 10083-2), carbon content range 0.27–0.35%; medium-carbon steel, designation 1.0503 (PN-EN 10083-2), carbon content range 0.42–0.50%; and high-carbon steel, designation 1.0601 (PN-EN 10083-2), carbon content range 0.57–0.65%. Mechanical properties of the tested steels are presented in Table 2 [56].

**Figure 8:**
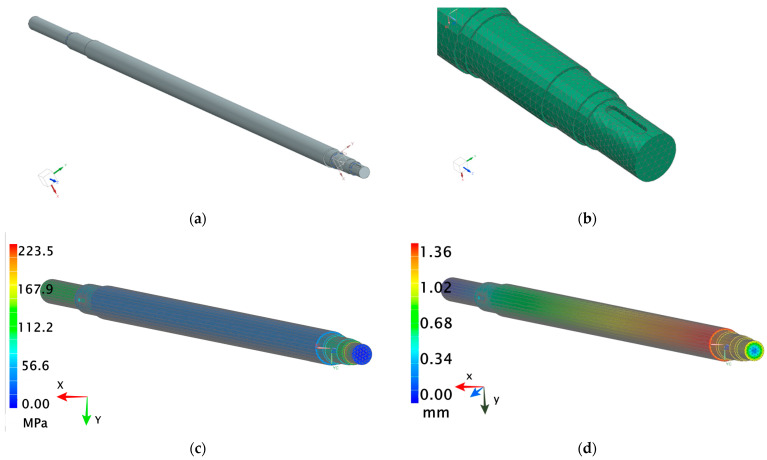
The model applied to FEM analysis (**a**); the used mesh (**b**,**c**) reduced stress (according to the von Mises criterion) distribution in the analyzed shaft; (**d**) displacement field.

The developed model of the shaft that failed allowed for a detailed analysis of the causes of damage that may have led to the failure, as well as the identification of potential design and material factors affecting the shaft’s durability (Figure 8a). A finite element mesh was applied to the shaft model, enabling analysis using the finite element method (FEM), with particular focus on the distribution of stresses and strains in various areas of the shaft (Figure 8b). The geometric model of the shaft was automatically meshed using four-node tetrahedral solid finite elements. Due to meshing, approximately 1,380,000 elements were created for the solid shaft model (the average element size was 4 mm). The use of smaller finite elements generated similar simulation results (the difference in stress values was less than 5%). It should be noted that the mesh density in specific places of the solid and hollow shafts was greater and the average size of the elements was equal to 1 mm. The necessary constraints included removing the degrees of freedom in the nodes on the shaft surface at the location of the rolling bearings (all displacements and two rotations were fixed). It was important that rotation around the axis of the shaft remained free.

Although the finite element analysis was performed on a hollow shaft, the holes at the ends of the shaft are not visible in the figures, as sunk journals were placed at these locations. This approach allows for proper representation of supports and torque transmission while maintaining clarity in the presented diagrams.

The shaft was subjected to an applied torque of 3800 Nm, representing the operational load during service. The shaft was fixed on one side, while a coupling was applied on the other side, and additionally the shaft was supported by bearings.

The stress analysis was carried out based on the Huber–Mises–Hencky hypothesis (commonly known as the von Mises criterion), which allows for the determination of equivalent (reduced) stresses representing the measure of an equivalent complex load. These stresses account for the simultaneous action of principal stresses in different directions and serve as the basis for assessing the potential onset of material yielding.

As a result of the numerical analysis, it was found that the highest stress occurred at the mounting point of the upper bearing (Figure 8c), which was crucial for further assessment of the shaft’s structural strength, whereas Figure 8d shows the displacements of the shaft. In the context of continued operation, the critical limitation proved to be the inability to modify the design of the bearing housing, which was an obstacle to modification of the load distribution system. To improve the durability of the shaft structure from the point of view of the most loaded areas, steel with increased carbon content was chosen for further simulation. When selecting the steel grade, the economic aspect was also considered by comparing the prices of considered carbon steel grades: 1.0501, 1.0503, and 1.0601. The average prices were: steel 1.0501, EUR 0.82/kg; steel 1.0503, EUR 1.29/kg; and steel 1.0601, EUR 1.64/kg. Considering the shaft’s weight of 152.1 kg, the total material cost for each steel was EUR 125 (1.0501); EUR 196 (1.0503) and EUR 249 (1.0601) [57]. Because of the large mass of the shaft and the significant increase in production costs, especially when choosing steel 1.0601, a decision was made to consider an alternative solution involving a redesign of the shaft. The goal was to design a more durable shaft without increasing the cost of its production.

**Figure 9:**
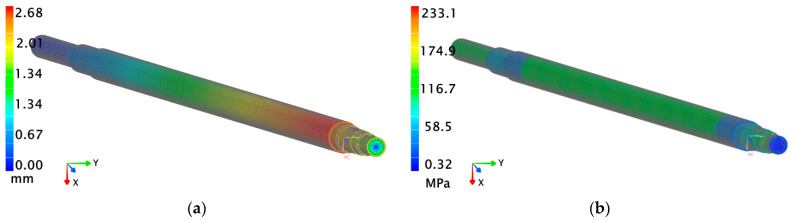
Analysis of the hollow shaft with a wall thickness of 5 mm: (**a**) displacement values; (**b**) reduced stresses values.

In the second stage of the analysis, using the finite element method (FEM), the focus was placed on developing various design variants of a hollow shaft in order to assess its behavior under load. Hollow shafts are widely used in aerospace, transportation, and other fields [58,59,60]. As part of this stage, three design variants of the hollow shaft were developed, differing in wall thickness (5 mm, 10 mm, and 15 mm). For each variant, simulations were carried out using three different steel grades: 1.0501, 1.0503, and 1.0601. The conducted numerical analysis took into account several key mechanical parameters, including displacement and reduced stresses. The value of the reduced stress allows for an assessment of a material’s strength under complex loading conditions, enabling better prediction of potential crack initiation sites and plastic deformation zones. The analysis of displacement parameters was carried out to examine the degree of deflection and potential deformation of the shaft under external loads. In turn, the distribution of reduced stresses enabled the assessment of the variation and intensity of stresses throughout the material volume, which is crucial for a shaft’s durability and its ability to withstand specified loads without losing structural integrity. Additionally, each configuration was evaluated in terms of material costs, allowing for the integration of strength and economic analysis. Although a hollow shaft with greater wall thickness made from higher-strength steel has the highest manufacturing cost, it provides significantly greater resistance to dynamic and fatigue loads in this case. Redesigning the paint mixer shaft by implementing a hollow shaft with a wall thickness of 5 mm enabled a significant reduction in the overall structure’s weight. This modification resulted in a 53% weight reduction, corresponding to a decrease of 70.4 kg in comparison to the solid shaft version. However, the use of a shaft with reduced wall thickness had unacceptable consequences in terms of deformation. The analysis showed that the maximum shaft displacement increased from the initial value of 1.3 mm to 2.6 mm, indicating a twofold increase in deflection under operating loads (Figure 9a). Additionally, an increase in reduced stresses by 10 MPa was also determined (Figure 9b).

An increase in stress values may indicate a higher risk of fatigue damage within the shaft cross section, particularly in areas of stress concentration. In the context of the shaft’s long-term durability, the rise in stress levels may necessitate the use of materials with even higher strength (e.g., alloy steel) or reinforcement of the structure in critical areas.

As a result of the FEM analysis of the second design variant—a hollow shaft with a wall thickness of 15 mm—a significant improvement was observed. The analyzed parameters were similar to those obtained for the solid shaft (displacement 1.3 mm), indicating that this design provided adequate stiffness. However, the consequence of these changes was a substantial increase in shaft weight from 70.4 kg to 116.4 kg (an increase of 65%). Greater weight not only adds extra load to the structure but also leads to higher material costs. The material costs for the 15 mm wall thickness shaft for the steel grades were EUR 95 (1.0501), EUR 150 (1.0503), and EUR 190 (1.0601).

**Figure 10:**
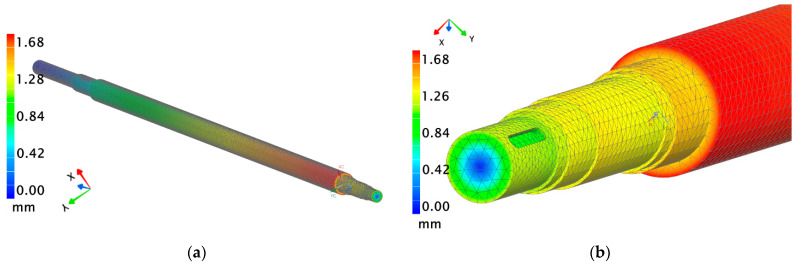
FEM analysis of the hollow shaft with 10 mm wall thickness: (**a**) displacement values; (**b**) location of maximum displacement.

Due to the increase in cost and weight, it was decided to consider one more design variant of the hollow shaft, this time with a wall thickness of 10 mm. In the case of using a hollow shaft with a 10 mm wall thickness, the strength parameters were only slightly worse than those determined in the 15 mm-wall-thickness variant (maximum displacement 1.7 mm; maximum stress 232 MPa). This means that the 10 mm thick shaft still provides adequate stiffness and strength for use in the paint mixer, showing only minimal differences in parameters such as displacement and reduced stress compared to the 15 mm variant. Figure 10 present the analyzed displacement and reduced stress parameters.

As a result of reducing the wall thickness to 10 mm, the drive shaft’s weight decreased from 116.4 kg to 99.7 kg (by 14%). The reduction in mass is expected to have a positive impact on the mixer’s operational dynamics, leading to lower loads and potentially extending the lifetime of the drive system, while also reducing electrical energy consumption. An additional benefit is the reduction in material costs, which for the three analyzed steel grades amounts to EUR 82 (1.0501), EUR 128 (1.0503), and EUR 164 (1.0601). Thanks to this compromise, it was possible to design a structure with mechanical properties similar to the 15 mm-wall-thickness variant while simultaneously reducing the weight and therefore the material costs. The 10 mm variant thus represents a potentially optimal solution, combining the required structural strength with the goal of maintaining material costs at a consistent level.

## 4. Conclusions

1. In less heavily loaded structures, the use of carbon steel as the material for the drive shaft is entirely sufficient and economically justified. The achieved service life of the analyzed paint mixer drive shaft, amounting to 20 years, should be considered satisfactory.
2. The regeneration of the drive shaft by weld-surfacing enabled its further operation for a relatively short period (3 years) and led to a change in the nature of its failure due to microstructural changes in the steel caused by thermal effects.
3. The cause of damage to the drive shaft prior to regeneration was chipping/wear of the outer surface, whereas the direct cause of failure after regeneration was cracking originating from initiation points in the outer zone of the surface layer. Therefore, the quality of the layer added during surfacing is a key factor in this case.
4. The failure of the shaft was also influenced by the excessive hardness of the added material (325 HV 0.1). Although no further surface abrasion wear was observed, the multisite, circumferential cracking mechanism suggests the excessive brittleness of the outer layer as a reason for destruction.
5. The radial expansion direction of the observed cracks indicated that the drive shaft experienced fatigue failure during service and the main reason for failure was rotation-bending fatigue.
6. The hardness of the core of the drive shaft was found to be typical for the standard value during the mechanical property test, which was due to the pearlitic–ferritic structure (with 60% pearlite).
7. The heat-affected zone formed as a result of the weld-surfacing process exhibited hardness only slightly lower than that of the newly added layer (by approximately 50 HV0.01) and consisted of very fine pearlite.
8. Finite element analysis showed that the drive shaft lifetime can be extended by using different materials: steel with higher carbon content or hollow shaft construction. The use of a hollow shaft can lead to a reduction in initial weight by up to 14%, which should result in a significant extension of the analyzed structure’s service life and enable its production from a higher-grade steel without increasing material costs.

## Figures and Tables

**Table 1:** Chemical composition of the tested drive shaft.

Chemical Composition of Steel, %								
	C	Si	Mn	P	S	Cr	Ni	Mo
PN-EN 10083-2	0.32–0.39	max 0.4	0.5–0.8	max 0.045	max 0.045	max 0.4	max 0.4	max 0.1
	C	Si	Mn	P	S	Cr	Ni	Mo
Analysis	0.34	0.25	0.64	0.025	0.020	0.25	0.20	0.1

**Table 2:** Mechanical properties of tested steel grades [56].

Mechanical Properties of Tested Steel			
Steel Grade	Yield Strength, MPa	Tensile Strength, MPa	Torsional Strength, MPa
1.0501	300–450	450–600	220–250
1.0503	450–600	600–800	550–700
1.0601	550–700	700–850	650–800

## Data Availability

The original contributions presented in this study are included in the article. Further inquiries can be directed to the corresponding author.
